# Performance in the Yo-Yo Intermittent Recovery Test May Improve with Repeated Trials: Does Practice Matter?

**DOI:** 10.3390/jfmk8020075

**Published:** 2023-06-06

**Authors:** Erika Zemková, Martin Pacholek

**Affiliations:** 1 Department of Biological and Medical Sciences, Faculty of Physical Education and Sport, Comenius University in Bratislava, 81469 Bratislava, Slovakia; 2 Faculty of Health Sciences, University of Ss. Cyril and Methodius in Trnava, 91701 Trnava, Slovakia; 3 Health and Physical Education Department, Prince Sultan University, Riyadh 12435, Saudi Arabia; mpacholek@psu.edu.sa

**Keywords:** distance covered, learning effect, performance level, Yo-Yo Intermittent Recovery Test

## Abstract

The Yo-Yo Intermittent Test is frequently used to monitor changes in athletes’ performance in response to different interventions. However, the question remains as to whether, and to what extent, retakes of this test would contribute to these changes. This case study sought to determine the magnitude of practice effects, involving test repetition, on performance in the Yo-Yo Intermittent Recovery Test. A recreational soccer player performed four attempts of the Yo-Yo Intermittent Recovery Test—Level 1 (YYIR1) with a week’s rest in between. The same participant repeated this test protocol (four attempts of the YYIR1) again after six months. Changes in distance covered, level achieved, maximal oxygen uptake, and heart rate between the first and last attempt were assessed. The smallest worthwhile change (SWC), the coefficient of variation (CV), and the 2CV were calculated to identify a trivial, a possibly meaningful, and a certainly meaningful change in YYIR1 performance. The distance covered in the first set of measurements increased from 1320 m to 1560 m (15.4%), which corresponds to a 4.6% increase in the level achieved (from 16.6 to 17.4). Similarly, the distance covered in the second set of measurements increased from 1280 m to 1560 m (17.9%), which corresponds to a 5.5% increase in the level achieved (from 16.5 to 17.4). The participant’s performance changes fell outside of the SWC and the CV, but not the 2CV during both sets of measurements. These improvements in YYIR1 performance may be ascribed to practice with repeated attempts of the test by improving running technique at the turning point and/or by simply increasing the linear speed. This fact should always be kept in mind when interpreting the effects of training. Practitioners should differentiate between practice effects associated with repeated test execution and adaptation induced by conducting sport-specific training.

## 1. Introduction

Since the introduction of the Yo-Yo Intermittent Tests to assess an individual’s ability to repeatedly perform intense exercise [1,2], they have been frequently used in practice. The Yo-Yo Intermittent Recovery Test is the most popular test used in team sports, such as football/soccer, e.g., [3,4,5], futsal [6,7,8], basketball [9,10], handball [11,12,13], hockey [14], rugby [15,16,17,18], and volleyball [19].

The Yo-Yo Intermittent Recovery Test—Level 1 (YYIR1) consists of two 20 m shuttle runs at increasing speeds interspersed with 10 s periods of active recovery until the individual cannot keep the required speed at a particular level [2]. During the test, the individual relies more on the aerobic system than the anaerobic system, which mostly determines YYIR1 performance. This may be demonstrated by a strong correlation between the YYIR1 distance covered in the test and maximal oxygen uptake (VO_2_max) [20,21], although it should be noted that some authors found only a moderate relationship [22]. The anaerobic contribution is greater during the Yo-Yo Intermittent Recovery Test—Level 2 (YYIR2) [21], where VO_2_max is only moderately related to YYIR2 performance [20,21,22]. As such, these tests have been used to evaluate the effectiveness of exercise training that stimulates the adaptation of aerobic and anaerobic capabilities. According to Castagna [23], the YYIR1 can be regarded as an aerobic–anaerobic soccer-specific field test. Although shuttle-running protocols using time to exhaustion or distance covered simulate the physiological demands of soccer, they are not valid measures of soccer performance because they do not include measures of soccer-specific skills [24].

However, the YYIR also represents a high-intensity exercise, which can induce beneficial physiological responses. Indeed, a six-week training program, involving the use of the YYIR1 twice a week, significantly increased the distance covered in this test (from 230 m to 403 m), and power in the Wingate anaerobic test in sedentary female university students [25].

In addition to adaptation to such an intervention, a learning effect may also play a role in improving the YYIR performance. The subject may become more proficient with increased experience. For instance, a potential learning effect was observed in the heart rate obtained during the 6 min YYIR1, with lower levels of variability found between the second and third trials [26]. At least one practice session before the experiment commences has been recommended so that the participants could become familiarized with the testing protocol [24]. However, familiarization may not be necessary for achieving excellent test–retest reliability, as shown in the case of the 30–15 Intermittent Fitness Test, although it might increase the test reliability [27]. In general, time trials with a coefficient of variation (CV) < 5% are more reliable than time-to-exhaustion protocols with a CV > 10% [24]. In addition to their reliability and validity, they should be sensitive in revealing small time course changes in athlete performance. This is more important for recreational physically active individuals than competitive athletes, such as soccer players, who may have more experience with shuttle running to exhaustion. The question, however, remains as to whether, and to what extent, retakes of the YYIR1 contribute to adaptive changes induced by interventions in recreational and competitive athletes. This case study evaluates the effect of practice on performance in the Yo-Yo Intermittent Recovery Test through repeated trials of this test.

## 2. Materials and Methods

### 2.1. Participant

A recreationally trained male soccer player (age: 42 years, height: 177 cm, body mass: 81.5 kg, BMI: 26 kg/m^2^) volunteered to participate in the study. The inclusion criterium included a healthy, physically active individual with no experience with the Yo-Yo Intermittent Recovery Test. He underwent a medical examination led by a physician before the experiment. No musculoskeletal problems were reported and no medically invasive procedures for lower limbs were previously performed.

The participant provided written informed consent to participate in the study. He was informed about the main aim of the study and related procedures. The procedures followed were in accordance with the ethical standards on human experimentation stated in compliance with the 1964 Helsinki Declaration and its later amendments.

### 2.2. Experimental Protocol

**Figure 1:**
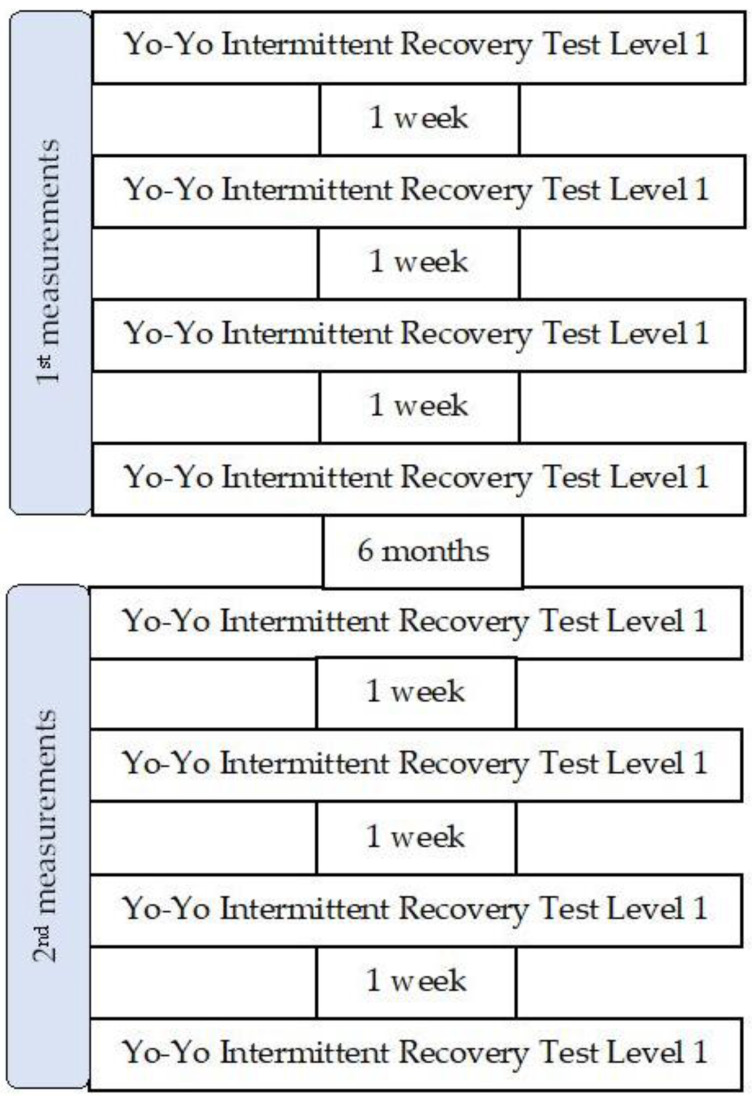
Flowchart of the experimental protocol.

Two sets of measurements, with a six-month break between them, were performed in order to conduct the experiment on similar performance levels during both occasions (Figure 1). During the 6-month break period, the participant participated in regular physical activities, similar to before the first set of measurements (team sports practice 3 times per week plus strength training). Each set consisted of four measurements separated by a one-week rest period. The participant was asked to avoid intense exercise 48 h preceding and during the study. The experiment was carried out under the same standardized conditions (e.g., in the same location at the same time).

Prior to the experiment, the participant was given instructions about the test. Measurements were taken on the same day of each experimental week. Each measurement started with a warm-up consisting of two minutes of jogging, two minutes of dynamic stretching, and two minutes of athletic running drills. After warming up, he underwent the Yo-Yo Intermittent Recovery Test—Level 1 (YYIR1), consisting of 2 × 20 m shuttle runs repeated at increasing speeds, with a 10 s recovery period between each shuttle, until exhaustion. He was asked to keep in time to the recording and complete the full 20 m run. He started with a foot behind the line and began to run when instructed by the audio recording. He turned when signaled by the recorded audio beep and returned to the starting point. He was instructed not to start running early, to run the complete distance, and to reach each line before or in time with the recording. There was a 10 s active recovery period (walking or jogging) between every 40 m run. The running speed increased at regular intervals. The starting speed of 10.0 km/h increased to 12 km/h, 13 km/h, and then by 0.5 km/h. The participant’s score was the level and total distance covered in the last complete successful shuttle. The VO_2_max (mL·kg^−1^.min^−1^) was also estimated using the formula of Bangsbo et al. [2]. Heart rate was monitored throughout the test using Polar Team Pro (Polar Electro, Kempele, Finland).

### 2.3. Data Analysis

The percentage change was calculated as the difference between the value of the last test and the value of the first test, which is then divided by the value of the last test and multiplied by 100. Furthermore, the smallest worthwhile change (SWC) was calculated as the standard deviation of the YYIR1 multiplied by an effect size of interest, i.e., 0.2 [28]. Furthermore, the coefficient of variation (CV) was calculated as follows: (standard deviation/mean) × 100. In addition to the SWC and the CV, the 2CV was calculated to provide the greatest certainty in YYIR1 performance changes.

## 3. Results

The distance covered in the YYIR1 during the first set of measurements increased from the first to the fourth attempt by 15.4%, which corresponds to a 4.6% increase in the level achieved (Table 1). Similarly, the distance covered in the YYIR1 during the second set of four measurements increased by 17.9%, which corresponds to a 5.5% increase in the level achieved (Table 2). The SWCs for the distance covered in the first and the second sets of measurements were 20.7 m and 26.5 m, respectively; the CVs were 7.2% and 9.1%, respectively (i.e., 103.3 m and 132.7 m, respectively); and the 2CVs were 206.6 m and 265.3 m, respectively.

## 4. Discussion

In the first set of measurements, the distance covered increased by 80 m (four 20 m shuttles) during the first trial, by 80 m (four 20 m shuttles) during the second trial, and by 80 m (four 20 m shuttles) during the third trial. In total, it has increased by 240 m (twelve 20 m shuttles), which represents 15.4%.

In the second set of measurements, the distance covered increased by 160 m (eight 20 m shuttles) during the first trial and by 120 m (six 20 m shuttles) during the second trial, while there were no changes during the third trial. In total, it has increased by 280 m (fourteen 20 m shuttles), which is 17.9%.

These improvements may be ascribed to practice with repeated attempts at this test by simply increasing linear speed and/or improving the running technique at the turning point. Considering that most of the time during this test is spent conducting linear sprinting, enhancing linear speed very likely contributed to the higher level achieved. Thus, athletes who are linearly fast may have an advantage in changing the direction tasks [29,30]. Presumably, an enhancement of a participant’s 180° turning ability also played a role in increasing the distance covered with repeated attempts of the test. Changing the direction of movement involves decreasing the horizontal momentum, followed by rotation of the body while placing the foot ahead of the center of mass in order to produce horizontal braking and propulsive impulses, which increase with the running speed [31,32,33]. This requires the eccentric strength of the knee extensors to reduce velocity levels in the deceleration phase, isometric strength in the amortization phase, and concentric strength in the acceleration phase [34,35,36]. The training targeting improvement in the quality of the side-step cutting technique usually addresses the modification of kinetic and kinematic movement patterns, as well as physical characteristics that are responsible for braking the movement and rapid force production during re-acceleration [37,38,39]. For instance, a 6-week change in direction speed and technique modification training improved the 180° turning performance. Athletes were able to produce greater mean horizontal propulsive force magnitudes in shorter ground contact times; apply and orientate the penultimate foot contact braking force and final foot contact propulsive force more horizontally; and display greater pelvis rotation, smaller knee flexion ROM during final foot contact, and greater velocity reduction and peak hip flexion angles during penultimate foot contact [40]. Similarly, 10-week eccentric overload training has been found to improve kinetic parameters during a change in direction football tasks [37].

The enhancement of YYIR1 performance should be interpreted in the context of the minimal change that falls outside the measurement error. The YYI tests are generally considered reliable [1,41] and sensitive [42]. The reported coefficients of variation (CVs) for the YYIR1 ranges from 4.9% [1], 7.3% [43], and 8.1% [2] up to 8.7% [20]. The application of these CVs to the distance covered by the participant in our study (1450 m on average) would represent suggestive meaningful improvements of 71.05 m (>three 20 m shuttles), 105.85 m (>five 20 m shuttles), 117.45 m (~six 20 m shuttles), and 126.15 m (>six 20 m shuttles). Taking into account CVs calculated in the present study for the first and the second sets of measurements (7.2% and 9.1%, respectively), the distance would be 103.68 m (~five 20 m shuttles) and 132.86 m (~six 20 m shuttles), respectively. Thus, changes in the distance covered in the range from 80 m to 160 m were greater than the estimated minimal detectable changes. It is therefore obvious that repeated attempts led to improvements in YYIR1 performance despite the fact that no exercise training was applied.

Based on the SWC, the participant should, on average, have to run a 23.6 m (20.7 m and 26.5 m, respectively) longer distance to demonstrate a meaningful improvement. So, the average distance covered of 1470.3 m (1460.7 m and 1479.9 m, respectively), or more, would be considered a meaningful change. It is evident that a change of about a one 20 m running distance is rather small. This is usually when the test has a more noise than the observed SWC [29]. In practice, the change in the individual’s performance needs be higher than the random variation associated with the test [44].

We can see that the CV in meters is much higher than the calculated SWC. Therefore, in this case, the CV would be much better to use for identifying a real change. So, when our data fall outside of the calculated variation (120 m on average, i.e., six 20 m shuttles), we can be confident of a real change. More specifically, the participant improved from trial to trial by 80 m during the first set of measurements, which is higher than the CV calculated for particular attempts (4.2%, i.e., 57.1 m; 3.9%, i.e., 56.2 m; and 3.7%, i.e., 56.2 m, respectively), which is 60 m on average. Similarly, the improvements by 160 m from the first to the second attempt and by 120 m from the second to the third attempt during the second set of measurements were also higher than the CVs calculated for these attempts (8.3%, i.e., 112.9 m; and 5.7%, i.e., 85.5 m, respectively). Despite the variation in the second set of measurements being higher than in the first set of measurements, the participant’s performance was above the CVs in both cases. Interestingly, he reached his maximum in the YYIR1 earlier in the second set of measurements than in the first set of measurements (during the third attempt), most likely due to familiarization with the test. To ensure that the threshold is large enough to account for a real change induced by the intervention, the CV needs to be doubled. Accordingly, the SWC, the CV, and the 2CV allow us to identify a trivial, a possibly meaningful, and a certainly meaningful change in YYIR1 performance. The participant’s performance changes induced only by practice of the test fall outside of the SWC and the CV, but not the 2CV (i.e., 114.2 m, 112.3 m, and 112.5 m, respectively, during the first set of measurements, and 225.8 m and 171.0 m, respectively, during the second set of measurements). However, further studies are needed to set the target for intervention studies that can provide a degree of certainty to which the changes are meaningful.

In general, a 15–35% improvement in the YYIR1 has been reported after 6 to 8 weeks of the aerobic high-intensity or speed endurance training [2]. To compare our findings with those obtained from intervention studies, data of age-matched referees were analyzed. They are, on average, 10 to 15 years older than their playing counterparts [45,46]. For instance, distance covered in the YYIR1 increased by 418 m, i.e., 23.7% (from 1345 m to 1763 m) after 12-week intense intermittent exercise training in male soccer referees with a mean age of 38 years [47]. Furthermore, distance covered in the YYIR1 also increased during long-term specific intermittent training (November, January, October, and March) in referees with a mean age of 37.8 years, specifically in elite referees by 141, 346, and 208 m, i.e., 9.8, 19.5, and 10.5%, respectively (from 1290 m to 1985 m) and in international referees by 15, 432, and 163 m, i.e., 0.86, 19.9, and 7.0%, respectively (from 1720 m to 2330 m) [48]. However, if we take into consideration the distance covered, their performance level was very likely much higher than that of our recreationally trained soccer player. This makes it somewhat difficult to compare with our results. Nevertheless, we can see that a 240–280 m increase in the distance covered over a course of four weeks without any specific training was, in some cases, higher than that induced by the intervention.

These changes in the YYIR1 induced by practicing the test were also greater than those observed after some training programs in recreational and competitive, but much younger (16–20 years on average) soccer players. For instance, a 6-week strength and soccer training led to a 147.7 m (i.e., 8.7%) increase in the distance covered (from 1547.7 m to 1695.4 m), whilst it was only a 3.1 m (i.e., 0.2%) increase after soccer training (from 1427.7 m to 1430.8 m) in recreational soccer players with a mean age of 18.5 years [5]. The distance covered also significantly improved after both 6-week combined core strength and small-sided games training by 179 m (from 1325 m to 1504 m) and small-sided games training by 149 m (from 1311 m to 1460 m) in young amateur soccer players with a mean age of 16.5 years [49]. A greater improvement in the distance covered was found after a 4-week high-intensity interval and resistance training compared to aerobic and strength training (468 m vs. 183 m) in young soccer players with a mean age of 17.8 years [50]. However, there were no significant differences in changes in the distance covered after a 3-week control period and 3-week intervention consisting of position-specific conditioning training (176.0 m vs. 178.7 m) in elite young soccer players with a mean age of 16.1 years [51].

Although the findings of these intervention studies cannot be compared with our results due to different experimental designs, experiment durations, and tested populations, it is evident that practice plays an important role in results obtained. Therefore, it may be premature to conclude that exercise training improves YYIR1 performance if similar or even greater improvements in the distance covered can be achieved after a simple repetition of this test. However, relevant experiments have yet to be performed to clarify the extent to which practice contributes to improvements in YYIR1 performance.

Findings of this case study underline the importance of gaining familiarity with the YYIR1 test protocol. Experience showed that besides appropriate pacing, using both legs equally during cutting maneuvers throughout the test may also contribute to better results. So, individuals who are familiar with the test procedure may achieve higher distance covered than those who do not have experience with this test. Therefore, familiarizing athletes with this test is highly recommended.

A limitation of the study is the small sample size consisting of only one male participant. For this reason, these findings cannot be generalized to the broader community based on this study alone. Therefore, further experiments on a larger sample size of athletes of different performance levels from various sports specializations have yet to be conducted to investigate to what extent practice helps to improve YYIR1 performance.

## 5. Conclusions

Performance in the Yo-Yo Intermittent Recovery Test improves after it is retaken. Specifically, the distance covered over four trials of this test separated by a one-week rest period increased by 15.4–17.9%, corresponding to a 4.6–5.5% increase in the performance level achieved. These performance changes fall outside of the smallest worthwhile change and a coefficient of variation (CV), but not the 2CV. These improvements in YYIR1 performance may be ascribed to practice with repeated attempts of the test by improving the running technique at the turning point and/or simply increasing linear speed. This fact has to be taken into account when the effect of training is evaluated. Practitioners should differentiate between practice effects associated with repeated test execution and adaptation induced by conducting sport-specific training.

## Figures and Tables

**Table 1:** Variables of the Yo-Yo Intermittent Recovery Test (Level 1) during repeated trials in the first measurement.

Level Achieved	Distance Covered (m)	VO_2_max (mL·kg^−1^·min^−1^)	Maximal Heart Rate (bpm)	Average Heart Rate (bpm)
16.6	1320	47.49	180	155
16.8	1400	48.16	187	163
17.2	1480	48.83	186	164
17.4	1560	49.50	181	162

**Table 2:** Variables of the Yo-Yo Intermittent Recovery Test (Level 1) during repeated trials in the second measurement.

Level Achieved	Distance Covered (m)	VO_2_max (mL·kg^−1^·min^−1^)	Maximal Heart Rate (bpm)	Average Heart Rate (bpm)
16.5	1280	47.15	182	152
17.1	1440	48.50	180	153
17.4	1560	49.50	185	157
17.4	1560	49.50	185	144

## Data Availability

The data are included.
